# Modification of Förster Resonance Energy Transfer Efficiency at Interfaces

**DOI:** 10.3390/ijms131115227

**Published:** 2012-11-19

**Authors:** Jörg Enderlein

**Affiliations:** III. Institute of Physics – Biophysics, Georg August University, Friedrich-Hund-Platz 1, D-37077 Göttingen, Germany; E-Mail: enderlein@physik3.gwdg.de; Tel.: +49-551-3913833; Fax: +49-551-397720

**Keywords:** Förster Resonance Energy Transfer, single-molecule fluorescence, single-molecule electrodynamics

## Abstract

We present a theoretical study on the impact of an interface on the FRET efficiency of a surface-bound acceptor-donor system. The FRET efficiency can be modified by two effects. Firstly, the donor’s electromagnetic field at the acceptor’s position is changed due to the partial reflection of the donor’s field. Secondly, both the donor’s and the acceptor’s quantum yield of fluorescence can be changed due to the interface-induced enhancement of the radiative emission rate (Purcell effect). Numerical results for a FRET-pair at a glass-water interface are given.

## 1. Introduction

Förster Resonance Energy Transfer or FRET [1] has become one of the most widely used fluorescence-spectroscopic tool in biophysical and biomedical research; see the reviews [2–6]. It allows for determining inter- and intramolecular distances of a few nanometers with sub-nanometer accuracy, and is routinely used for measuring intramolecular conformation and intermolecular interactions. In particular with the advent of single-molecule fluorescence spectroscopy, single-molecule FRET (smFRET) has become one of the most important toolbox of single-molecule spectroscopists [7,8].

Frequently, smFRET experiments are performed with the molecules immobilized on a surface [9,10]. This prevents the diffusion of the molecules out of the focal plane and allows for recording long-lasting photon bursts from one and the same molecule. Moreover, such measurements can be performed with a Total-Internal Reflection Fluorescence or TIRF microscope for maximizing the signal-to-background ratio. However, the proximity of an interface dividing to materials with different refractive indices does change the fluorescence emission behavior of fluorescent dyes. In particular, their radiative transition rate from the excited to the ground state is changed, which results in a changed fluorescence lifetime. Although this effect is small for interfaces between dielectric materials, the present paper aims at quantifying the potential impact of such electromagnetic effects on the accuracy of a smFRET measurement. To have precise knowledge about such surface-induced effects on the measurable FRET efficiency is important in the context of recent efforts to make smFRET measurements as exact and quantitative as possible [11–17].

In the past, several studies have been published that focus on the strong change of the FRET efficiency due to the coupling of the donor and acceptor fluorescence to the local density of states of the electromagnetic field as modified by the presence of a cavity [18] or a micro-resonator [19,20]. Here, we give a detailed study of this change due to the much weaker influence of an interface dividing two materials of different dielectric constants.

## 2. Theory

Before considering the problem of the FRET efficiency in the vicinity of an interface, we have to first briefly recall the classical theory of FRET, because the details of this derivation will be needed for a close understanding of the modifications induced by the presence of an interface that divides to half-spaces with different refractive index. In the second subsection, we will then study the change in acceptor excitation rate by the presence of an interface. In the third and last subsection, we will consider the interface-induced changes in fluorescence quantum yield of both the acceptor and the donor, which are important when calculating FRET efficiencies from the ratio of donor and acceptor intensities.

### 2.1. Classical Förster Theory

A light emitting molecule can be fairly well described by the classical picture of an oscillating electric dipole. Such a dipole is characterized by the charges *q* that oscillate, the oscillation amplitude and orientation described by the dipole vector **a**, and the oscillation circular frequency *ω*, which is also the frequency of the emitted light. The product of the charge *q* times the amplitude vector **a** is called the dipole moment **p***_d_* (or, more precisely, the amplitude of the dipole moment), *i.e*., **p***_d_* = *q***a**. When plugging such an oscillating electric dipole as a source into Maxwell’s equations, one obtains the electromagnetic field of the well-known Hertzian dipole emitter. In particular, the electric field amplitude reads

(1) $$E_{d}(r)=\left[\left(-\frac{k^{2}}{r}-\frac{3ik}{r^{2}}+\frac{3}{r^{3}}\right)e_{r}\;(e_{r}\cdot p_{d})+\left(\frac{k^{2}}{r}+\frac{ik}{r^{2}}-\frac{1}{r^{3}}\right)p_{d}\right]\frac{e^{ikr}}{n^{2}}$$

where **r** is the distance vector from the dipole to the observation point where the field is calculated, *r* is its modulus, *k* is the modulus of the wave vector, *k* = *nω/c*, with *c* being the vacuum speed of light, **e***_r_* is a unit vector pointing from the dipole towards the position **r**, and *n* is the refractive index of the medium. Here, and in all that follows, we omit the factor *e*^−^*^iωt^* describing the temporal evolution of the electric field. The far-field emission rate of such a dipole is given by

(2) $$S_{d}=\frac{1}{3}p_{d}^{2}k_{0}^{4}cn$$

where *k*_0_ is the length of the wave vector in vacuum (*k*_0_ = 2*π/λ*), with *λ* being the vacuum wavelength of light. When a second molecule (also assumed to be an electric dipole oscillator) is brought very close to the emitting molecule (the donor), the field contributions falling off proportional to *r*^−1^ and *r*^−2^ can usually be neglected if *kr* ≪ 1, and one has to take into account only the near-field component

(3) $$E_{d}(r)\approx [3e_{r}\;(e_{r}\cdot p_{d})-p_{d}]\frac{e^{ikr}}{n^{2}r^{3}}$$

The excitation rate of the second molecule (the acceptor) is given by the product of its absorption cross section *σ_a_* and the number of (virtual) photons per area per second coming from the donor. This photon flux is given by the electromagnetic energy flux density associated with the donor’s electromagnetic field divided by the energy per photon. This energy flux density, averaged over one oscillation period, is equal to the modulus of the Poynting vector or (*nc/*8*π*) · *E*^2^*_d_*, and the energy per photon is equal to *hc/λ*, where *h* is Planck’s constant. Furthermore, one has to take into account that the acceptor’s excitation rate is also orientation-dependent and proportional to the scalar product of its dipole vector **p***_a_* and the vector of the exciting electric field **E***_d_*. One minor complication comes in by the fact that when one measures absorption cross-sections *σ_a_* on ensembles of molecules, one averages over all possible dipole orientations, so that the absorption cross-section of a molecule with its dipole aligned with the electric field vector of the exciting field is three times larger than the measured value of *σ_a_*. Taking all that together, one arrives at the following expression for the acceptor’s excitation rate *k_e_*:

(4) $$k_{e}=(3\sigma_{a})\frac{nc}{8\pi }\frac{\lambda }{hc}|{\hat{p}}_{a}\cdot E_{d}{|}^{2}$$

where **p̂***_a_* is the unit vector along the acceptor’s dipole moment. Inserting the explicit expression for **E***_d_* yields

(5) ke=3σaλpd28πhn3r6∣3 (er·p^a) (er·p^d)-(p^a·p^d)∣2=3σaλpd28πhn3r6κ2

which also defines the so-called orientation factor *k*. If both donor and acceptor can freely rotate, one can average the orientations **p̂***_a_* and **p̂***_d_* of donor and acceptor dipole, resulting in *k*^2^ = 2*/*3.

The equation for *k_e_* still contains the *a priori* unknown donor dipole amplitude *p_d_*. It can be found by scrutinizing the total energy emission of the free donor. From a quantum mechanical point of view, the totally emitted energy is that of one photon, *hc/λ*. From a classical electromagnetic point of view, it is given by the energy emission rate of the donor times the mean *radiation-related* lifetime of the donor’s excited state (*i.e*., its lifetime in the absence of all non-radiative de-excitation channels), which is given by the inverse of the *radiative* transition rate *k_f;d_* from the excited to the ground state of the donor. The inverse of the sum of the radiative and the non-radiative transition rate, *k_nr;d_*, determines the observable excited state lifetime *τ_d_*, whereas the ratio of *k_f;d_* and *k_f;d_* + *k_nr;d_* define the donor’s fluorescence quantum yield *φ_d_* (*i.e*., the probability that the donor’s energy is given away electromagnetically, and not by intermolecular collisions *etc*.). Thus, *k*^−1^*_f;d_* is given by the ratio of the observable excited state lifetime *τ_d_* divided by the fluorescence quantum yield *φ_d_*. Setting both the quantum-mechanical and the classical-electromagnetic energy emission equal to each other, one obtains

(6) $$\frac{hc}{\lambda }=\frac{1}{3}p_{d}^{2}k_{0}^{4}cn\cdot \frac{\tau_{d}}{\varphi_{d}}$$

where we have used Equation 2 for the far-field emission rate *S_d_*. Thus, one finds

(7) $$p_{d}^{2}=\frac{3h\varphi_{d}}{2\pi k_{0}^{3}n\tau_{d}}$$

so that the acceptor’s excitation rate now reads

(8) $$k_{e}=\frac{9\sigma_{a}\varphi_{d}}{8\pi k_{0}^{4}\tau_{d}n^{4}r^{6}}\kappa^{2}$$

Up to this point, donor emission and acceptor excitation was assumed to happen at exactly one and the same wavelength. To obtain the actual acceptor excitation rate, one has to integrate the found expression over all wavelengths, taking into account the wavelength-dependence of the donor’s emission strength as given by its emission spectrum *F_d_*(*λ*), and of the acceptor’s excitation strength as given by its absorption spectrum *σ_a_*(*λ*). This leads to

(9) $$k_{e}=\frac{9\varphi_{d}\kappa^{2}}{8\pi {\left(2\pi \right)}^{4}\tau_{d}n^{4}r^{6}}\frac{\int d\lambda F_{d}(\lambda )\sigma_{a}(\lambda )\lambda^{4}}{\int d\lambda F_{d}(\lambda )}$$

where *F_d_*(*λ*)*/∫ dλF_d_*(*λ*) is the normalized emission spectrum of the donor, and where we have replaced *k*_0_ by 2*π/λ*. The so-called Förster radius is defined by

(10) $$R_{0}^{6}=\frac{9\varphi_{d}\kappa^{2}}{8\pi {\left(2\pi \right)}^{4}n^{4}}\frac{\int d\lambda F_{d}(\lambda )\sigma_{a}(\lambda )\lambda^{4}}{\int d\lambda F_{d}(\lambda )}$$

so that one finally finds

(11) $$k_{e}=\frac{1}{\tau_{d}}\;{\left(\frac{R_{0}}{r}\right)}^{6}$$

Taking into account the relation between absorption cross section *σ_a_* (in cm^2^) and molar extinction coefficient *ε* (in cm^−1^ M^−1^),

(12) $$\sigma_{a}=\frac{10^{3}\;\text{ln}\;10}{N_{A}}{ɛ}_{a}$$

where *N_A_* is the Avogadro–Loschmidt number, one finds the usual textbook expression for *R*_0_

(13) $$R_{0}=\frac{9000\;\text{ln}\;10}{128\pi^{5}N_{A}}\frac{\varphi_{d}\kappa^{2}}{n^{4}}\frac{\int d\lambda F_{d}(\lambda ){ɛ}_{a}(\lambda )\lambda^{4}}{\int d\lambda F_{d}(\lambda )}$$

Finally, we have to discuss the two most common methods of experimentally determining FRET efficiencies. The first one measures the fluorescence intensity of the donor and the acceptor upon donor excitation. For the sake of simplicity we assume that one can neglect direct excitation of the acceptor by the excitation light, and any bleed-through of donor emission into the acceptor detection channel. Also, the detection efficiencies for both donor and acceptor fluorescence are assumed to be the same. Then the observable fluorescence intensity from the donor in the presence and in the absence of the acceptor will be proportional to

(14) $$I_{da}\propto \frac{k_{f,d}}{k_{f,d}+k_{nr,d}+k_{e}}$$

and

(15) $$I_{d}\propto \frac{k_{f,d}}{k_{f,d}+k_{nr,d}}=\varphi_{d},$$

respectively, and that of the acceptor is proportional to

(16) $$I_{a}\propto \frac{k_{f,a}}{k_{f,a}+k_{nr,a}},\frac{k_{e}}{k_{f,d}+k_{nr,d}+k_{e}}=\frac{\varphi_{a}k_{e}}{k_{f,d}+k_{nr,d}+k_{e}}$$

Here, the *k_f_* and *k_nr_* are the radiative and non-radiative transition rates, respectively, and the additional subscript *d* refers to the donor and the subscript *a* to the acceptor. Accordingly, *φ_a_* is the fluorescence quantum yield of the acceptor. Thus, we find the textbook relation for the FRET efficiency *E*:

(17) $$E=\frac{I_{d}-I_{da}}{I_{d}}=\frac{I_{a}/\varphi_{a}}{I_{a}/\varphi_{a}+I_{da}/\varphi_{d}}=\frac{k_{e}}{k_{f,d}+k_{nr,d}+k_{e}}=\frac{R_{0}^{6}}{R_{0}^{6}+r^{6}}$$

where we have used Equation 11 and the fact that the free donor’s de-excitation rate is the inverse of its lifetime, *k_f;d_* + *k_nr;d_* = *τ*^−1^*_d_*. The second method is based on the measurement of the donor’s excited state lifetime. In the presence of the acceptor, this lifetime is given by

(18) $$\tau_{da}=\frac{1}{k_{f,d}+k_{nr,d}+k_{e}}$$

Thus, one finds a second relation for the FRET efficiency *E* as

(19) $$E=\frac{\tau_{d}-\tau_{da}}{\tau_{d}}=\frac{k_{e}}{k_{f,d}+k_{nr,d}+k_{e}}=\frac{R_{0}^{6}}{R_{0}^{6}+r^{6}}$$

### 2.2. Donor Electric Field above the Surface

**Figure 1:**
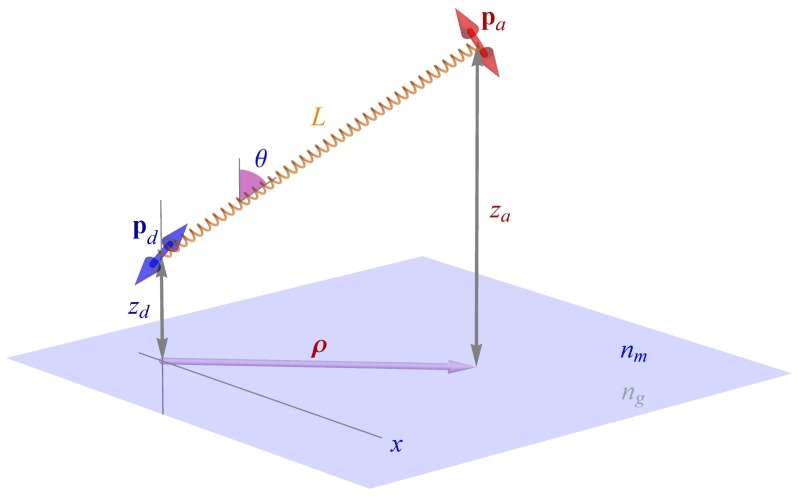
Geometry of the considered problem: A donor molecule with dipole moment *p_d_* is kept at a distance *z_d_* above a surface. An acceptor molecule with dipole moment *p_a_* is situated a fixed distance *L* away from the donor, the angle between the vertical and the connecting line is *θ*.

Consider the system depicted in Figure 1: A donor-acceptor pair (smFRET system) are coupled together by some rigid ruler keeping them a fixed distance *L* apart. One of the molecules (let us assume the donor) is kept at a distance *z_d_* from a surface; the half-space below that surface has the refractive index *n_g_*, and the half-space above the surface, containing the smFRET system, has refractive index *n_m_*. As before, both the donor and the acceptor are considered to be ideal electric dipole emitters/absorbers. Furthermore, we will assume that both donor and acceptor are free to rapidly rotate around their center positions so that one can average over donor and acceptor orientation when calculating FRET efficiencies. As will be seen, an extension of the theory beyond this assumption is straightforward but would introduce another four degrees of freedom. But we will assume that the connecting line between acceptor and donor keeps a fixed angle towards the vertical. For example, in case of a charged surface and a charged smFRET system, one of two limiting cases may be expected: either both molecules are adsorbed to the interface and *θ* = *π/*2 due to attractive electrostatic interaction, or one finds *θ* ~ 0 due to electrostatic repulsion between surface and smFRET system. Our results will also be applicable to the more general case of orientational flexibility of the donor-acceptor axis. One has then only to average over different orientations of this axis with an appropriate weight function that describes the chance that a particular orientation occurs.

**Figure 2:**
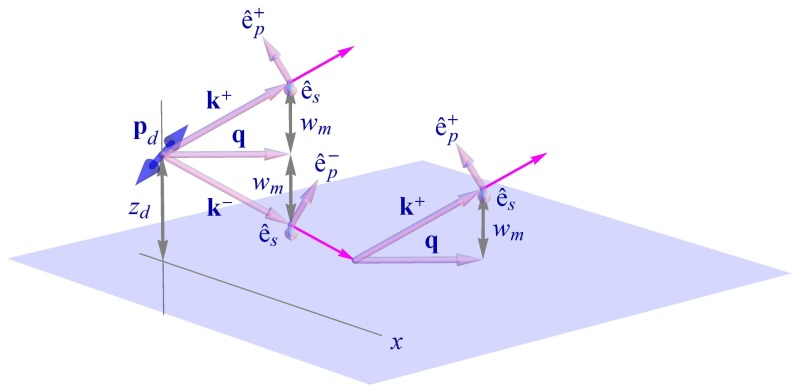
Visualization of the wave vectors and unit polarization vectors of the plane wave components as used in the Weyl representation of the donor electric field.

As we have seen in the preceding subsection, the energy transfer rate *k_e_* is proportional to the donor-generated local electric field strength at the position of the acceptor. Due to partial reflection at the interface, this field strength will be different from the situation without interface. For calculating this field strength, we start by expanding the electric field of the free donor into a Weyl representation [21] of plane waves (see Figures 1 and 2), which is the standard field representation when considering planar problems, similar to using Bessel function expansions for problems with cylindrical symmetry, or spherical harmonics expansions for problems with spherical symmetry:

(20) $$E_{d}(\rho ,z)=\frac{ik_{0}^{2}}{2\pi }\int \int \frac{dq}{w}\left[e_{p}^{\pm }\left(e_{p}^{\pm }\cdot p_{d}\right)+e_{s}\left(e_{s}\cdot p_{d}\right)\right]\;e^{iq\cdot \rho +iw|z-z_{d}|}$$

where **q** is the part of the wave vector parallel to the interface, and $w=\sqrt{k^{2}-q^{2}}$ its part perpendicular to the interface. The unit vectors **e**^±^*_p_* and **e***_s_* are pointing along the electric field polarization of *p*- and *s*-waves, respectively, as shown in Figure 2, where the plus sign applies for *z > z_d_*, and the minus sign for *z < z_d_*. The integration over **q** extends over the whole two-dimensional **q**-plane. The electric field as given in Equation 20 is the field of an oscillating electric dipole in free space with refractive index *n_m_* and yields, after explicit integration, the result of Equation 1.

In the presence of an interface, each plane wave component in the Weyl representation is partially reflected, adding to the total field strength in the upper half space. Using the standard, *q*-dependent Fresnel coefficients *r_p;s_* for the reflection of plane *p*- and *s*-waves at a planar interface, the reflected field can be written as

(21) $$E_{r}(\rho ,z)=\frac{ik_{0}^{2}}{2\pi }\int \int \frac{dq}{w}\left[r_{p}(q)e_{p}^{+}(e_{p}^{-}\cdot p_{d})+r_{s}(q)e_{s}(e_{s}\cdot p_{d})\right]e^{iw(z+z_{d})+iq\cdot \rho }$$

so that the total electric field strength in the upper half space is given by **E**′*_d_* = **E***_d_* + **E***_r_*. Whereas the integrals in Equation 20 can be calculated analytically, the integrals in Equation 21 have to be calculated numerically. The excitation efficiency of the acceptor by the field of the emitting donor is then proportional to the square of the scalar product between electric field amplitude and acceptor dipole moment, *i.e*., proportional to |**E**′*_d_*(***ρ****_a_, z_a_*) · **p***_a_*|^2^, where ***ρ****_a_* and *z_a_* are the lateral and vertical position of the acceptor, respectively, and **p***_a_* its excitation dipole moment. What we are interested in is a comparison of the FRET efficiency of our acceptor-donor system in free space with the same system attached to the surface. For the sake of simplicity, we will here compare both situations in the limit of rapidly rotating acceptor and donor orientation. As already mentioned, we could equally well consider particular fixed orientations of donor and acceptor, which would, however, add another four degrees of freedom without making the general effect of an interface on the FRET efficiency more clear. As will be seen from the calculations of the emission rate enhancement below, all the considered electrodynamic effects are strongly orientation-dependent, so that a fixed donor and/or acceptor orientation will lead, in general, to an even stronger modification of the FRET efficiency than in case of rapid rotational diffusion of donor and acceptor. Thus, we define the ratio

(22) $$Q(\rho_{a},z_{a})=\frac{{\langle |E_{d}^{\prime }(\rho_{a},z_{a})\cdot p_{a}{|}^{2}\rangle }_{\Omega }}{{\langle |E_{d}(\rho_{a},z_{a})\cdot p_{a}{|}^{2}\rangle }_{\Omega }}$$

which tells us how much the acceptor’s excitation rate of the surface-bound system is enhanced (*Q >* 1) or reduced (*Q <* 1) when compared with a measurement in free solution. Here, the angular brackets with subscript Ω indicate averaging over all orientations of donor and acceptor. Due to symmetry, the ratio *Q* depends only on the modulus *ρ_a_* = |***ρ****_a_*| of the lateral position vector, but not its two-dimensional orientation. Moreover, the problem is reciprocal: the ratio *Q* in Equation 22 remains the same when interchanging the positions of donor and acceptor. This can be directly checked by repeating the above calculations with interchanged positions.

### 2.3. Emission Rate above the Surface

Besides a change in the excitation intensity felt by the acceptor due to the back-reflection of the donor’s electromagnetic field, there is a second surface-induced aspect affecting the FRET efficiency. As we have seen in Section 2.1, the acceptor excitation rate *k_e_* is also proportional to the donor’s radiative transition rate *k_f;d_* = *φ_d_/τ_d_*. During the emission of the donor, the back-reflected electromagnetic field acts on the emitting donor itself, enhancing or inhibiting its radiation and thus changing its radiative transition rate. This is also called the Purcell effect after Edward Mills Purcell who first pointed it out in 1946 [22]. When the reflected field **E***_r_* acts on the oscillating dipole, the dipole performs the average work per time of (see [23])

(23) $$W=\frac{\omega }{2}p_{d}\cdot I(E_{r})$$

which adds an additional term to the energy loss per time exhibited by the dipole. Thus, the emission rate is changed to

(24) $$S_{d}^{\prime }=S_{d}+W$$

so that the relative change *γ* in emission rate alias radiative transition rate is given by

(25) $$\gamma (z_{d})=\frac{k_{f,d}^{\prime }}{k_{f,d}}=\frac{S_{d}^{\prime }}{S_{d}}=1+\frac{3\omega }{2{cnp}_{d}k_{0}^{4}}{\langle {\hat{p}}_{d}\cdot I(E_{r})\rangle }_{\Omega }$$

where we have again averaged over all possible orientations of the emission dipole, **p̂***_d_*. The factor *γ_d_* is the interface-induced change of the radiative transition rate of the donor. An identical relation holds also for the emission rate of the acceptor, where one has only to exchange all subscripts *d* by *a*. If one can neglect the optical dispersion of the materials in the upper and lower half spaces, then the resulting factor *γ* is identical for both donor and acceptor if they are at the same distance from the surface. Knowing both the factor *Q* and *γ*, the interface-modified acceptor excitation rate is given by

(26) $$k_{e}^{\prime }=\gamma Qk_{e}.$$

The modified radiative emission rate changes also the quantum yield of both the donor and the acceptor, according to the formula

(27) $$\varphi^{\prime }=\frac{k_{f}^{\prime }}{k_{f}^{\prime }+k_{nr}}=\frac{\gamma k_{f}}{\gamma k_{f}+k_{nr}}=\frac{\gamma \varphi }{\gamma \varphi +1-\varphi }$$

where we have omitted the subscript *d* or *a*.

### 2.4. FRET Efficiency above the Surface

When calculating the FRET efficiency *E* by the intensity ratio according to Equation 17, two scenarios are possible. If one uses the interface-modified values of the fluorescence quantum yields of both donor and acceptor, then the interface-modified FRET efficiency is given by

(28) $$E^{\prime }=\frac{I_{a}/\varphi_{a}^{\prime }}{I_{a}/\varphi_{a}^{\prime }+I_{da}/\varphi_{d}^{\prime }}=\frac{\gamma QE}{\gamma QE+(\gamma \varphi_{d}+1-\varphi_{d})(1-E)}$$

However, if one uses the quantum yield values for the dyes in free solution, the calculated FRET efficiency is

(29) $$E^{\prime }=\frac{I_{a}/\varphi_{a}}{I_{a}/\varphi_{a}+I_{da}/\varphi_{d}}=\frac{\gamma QE}{\gamma QE+(\gamma \varphi_{a}+1-\varphi_{a})(1-E)}$$

which becomes identical to Equation 28 only if *φ_a_* = *φ_d_*. Similarly, when using the lifetime-based Equation 19 for determining the FRET efficiency, one can either use the lifetime value *τ_d_* of the donor in free solution, or the interface-modified value *τ*′*_d_* = *τ_d_/*(*γφ_d_* + 1 −*φ*′*_d_*). When using *τ*′*_d_*, one recovers Equation 28, and when using *τ_d_*, one finds

(30) $$E^{\prime }=\frac{\gamma QE+(\gamma -1)\varphi_{d}(1-E)}{\gamma QE+(\gamma \varphi_{d}+1-\varphi_{d})(1-E)}$$

## 3. Results and Discussion

**Figure 3:**
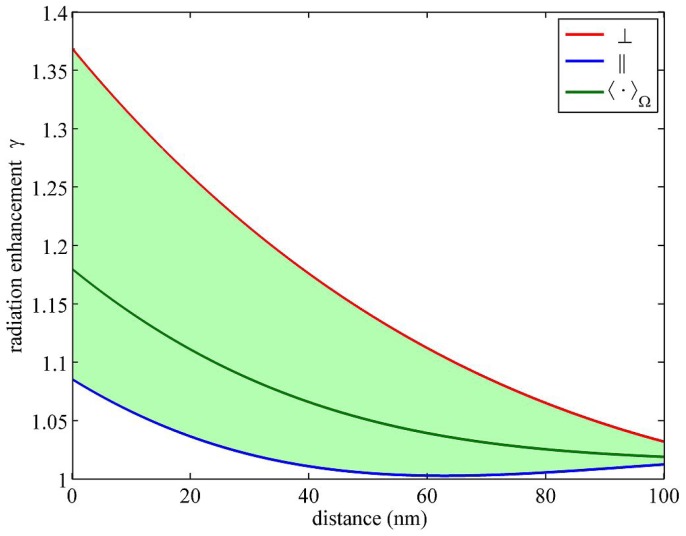
Radiation enhancement *γ* as a function of the distance of an emitter in water from a glass surface.

We first performed calculations of *γ*, *Q*, and *E*′ for a glass-water interface, where the refractive index of glass was set equal to 1:52 and that of water equal to 1:33. All calculations where done for an assumed emission wavelength of 550 nm. Figure 3 shows the distance dependence of the radiation enhancement, *γ*. As can be seen, close to the interface (a few dozen nanometers), *γ*does not change significantly, reaching a maximum value of 1.18 on the surface (averaged over all possible dipole orientations). For the sake of completeness, the figure shows also the extreme values of *γ* for horizontal (‖) and vertical (⊥) emission dipole orientation. An identical figure is obtained for the radiation enhancement of an acceptor, where the *x*-axis has only to be rescaled by the ratio of the acceptor’s to the donor’s emission wavelength. Taking into account the dependence of the fluorescence quantum yield on the enhancement factor *γ*, Equation 27, we find for the relative change in quantum yield Δ*φ/φ* = (1 − *φ*), Δ*γ/γ* so that a 18% change in the radiative rate *γ* for a fluorophore with 50% fluorescence quantum yield translates into an approximately 9% change in quantum yield. Of course, the effect becomes smaller with increasing value of quantum yield.

**Figure 4:**
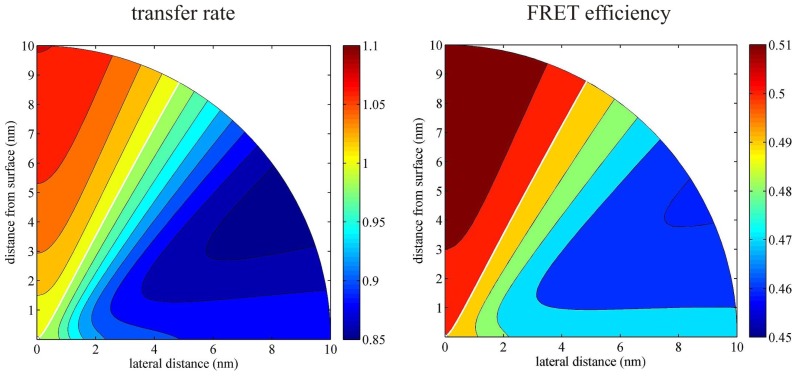
Enhancement *Q* of the energy transfer rate (left) and the FRET efficiency (right) as a function of the relative position of the acceptor (donor) if the donor (acceptor) is positioned directly on a glass surface (*n* = 1.52). For the FRET efficiency calculations (right panel), it was assumed that its value in free solution is 0.5 and that the quantum yield of the fluorophores is 1.0.

Next, Figure 4 presents the dependence of both the factor *Q*(*ρ_a_; z_a_*) and the FRET efficiency *E*′ as a function of lateral (*ρ_a_*) and vertical (*z_a_*) distance between donor and acceptor, respectively, where it is assumed that the donor sits directly on the surface at *ρ_d_* = 0. As was already mentioned, an identical figure is obtained when exchanging the positions of donor and acceptor.

The *Q*-factor varies between 0.87 and 1.06, depending on the orientation of the donor-to-acceptor axis. For example, for a donor-acceptor distance of 10 nm, if the donor-acceptor connecting line is close to the surface, *Q* is close to its minimum of 0.87, whereas if this axis is perpendicular to the surface, it reaches its maximum value of 1.06. When using Equation 28 or 29 for calculating the FRET efficiency, then the relative change in *E* is given by

(31) $$\frac{\Delta E}{E}=(1-E)\frac{\Delta Q}{Q}+(1-E)(1-\varphi )\frac{\Delta \gamma }{\gamma }$$

where *φ* = *φ_d_* for Equation 28, and *φ* = *φ_a_* for Equation 29. Thus, at midpoint FRET efficiency of 0.5, the relative change Δ*E/E* is half that of Δ*Q* (*Q* = 1) for dyes with quantum yield of 1.0, and the FRET efficiency is only changed between −7% and +3%. For quantum yield values of 0.5, the relative change in FRET efficiency lies between −2.5% and 4.5%. Thus, although the energy transfer rate *k_e_* can vary by up to 13%, and the radiative emission rate by up to 18%, the resulting variation in FRET efficiency is much smaller due to the intricate dependence of *E* on both these factors.

**Figure 5:**
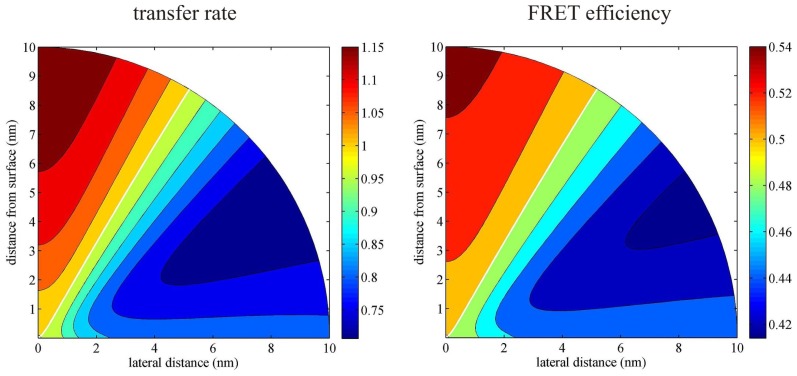
Enhancement *Q* of the energy transfer rate (left) and the FRET efficiency (right) as a function of the relative position of the acceptor (donor) if the donor (acceptor) is positioned directly on the sapphire surface (*n* = 1:78). For the FRET efficiency calculations (right panel), it was assumed that its value in free solution is 0.5 and that the quantum yield of the fluorophores is 1.0.

In recent years, objectives with N.A.-values of 1.65–1.7 have become available and are used for single-molecule detection and spectroscopy experiments, because they provide exceptional high light collection efficiency and enable efficient TIR illumination with very small penetration lengths of the evanescent field. When using these objectives, one needs special sapphire cover slides and high-index immersion oils with a refractive index of 1.78. We repeated all the above calculations for checking how such a high refractive-index material will influence the smFRET measurement. For a fluorophore close to the sapphire/water interface, the radiative emission rate is now enhanced by 45% (averaged over all dipole orientations) in contrast to the 18% for the glass/water interface. Figure 5 shows the results for both the transfer rate *Q* and the FRET efficiency *E*′ for the same geometry and distances as used in the calculations for the glass/water interface. The enhancement factor *Q* for the transition rate varies now between 0.71 and 1.2, and the resulting FRET efficiency varies between −17% and +9% for dyes with quantum yield 1.0, and between −9% and +17% for dyes with quantum yield 0.5. Thus, although the impact of a glass/water interface on the FRET efficiency is rather moderate, it is no longer negligible for a sapphire/water interface.

There are two possible and straightforward ways to experimentally test the predictions made in this paper. The simplest way would be to compare FRET measurements of the same system in free solution, and when it is bound to a surface. However, a problem might be that other factors like the different nano-environment close to a surface or the binding chemistry used for surface attachment may also influence the measured FRET efficiency. A more elegant way would be to use a short and rigid DNA double strand with donor and acceptor at both ends as the smFRET system, and to bind it with one end to the surface. If the surface is prepared in such a way that it carries a negative net charge, one can assume that the negatively charged DNA double strand will adopt an orientation orthogonal and away from the surface, whereas for a positively charged surface, the double strand will adsorb to the surface and thus lie flat on it. As can be seen from Figures 4 and 5, one expects an opposite effect on the FRET efficiency for both cases: For an orientation perpendicular to the surface, the FRET efficiency is enhanced, whereas for an orientation parallel to the surface, it is decreased. At least for a sapphire substrate, the difference between both situations (±10%) should be easily observable.

## 4. Conclusions

In the present paper, we have given a complete theoretical analysis of the impact of interface-induced changes in the donor’s electric field as well as the radiative transition rates of both donor and acceptor on the FRET efficiency. Although the donor’s electric field as well as the radiative transition rates can change by more than ten percent, the resulting impact on FRET efficiency for a glass/water interface is rather moderate. This is, however, no longer true when measurements are performed on high refractive-index substrates such as sapphire, which is used in some single-molecule experiments. Finally, we have given some indications of how to experimentally measure the predicted effects.

## References

[1] Förster T. (1948). Zwischenmolekulare energiewanderung und fluoreszenz. Ann. der Physik.

[2] Clegg R.M. (1992). Fluorescence resonance energy transfer and nucleic acids. Methods Enzymol.

[3] Clegg R.M. (1995). Fluorescence resonance energy transfer. Curr. Opin. Biotechnol.

[4] Clegg R.M., Wang X.F., Herman B. (1996). Fluorescence Resonance Energy Transfer (FRET). Fluorescence Imaging Spectroscopy and Microscopy.

[5] Jares-Erijman E.A., Jovin T.M. (2003). FRET imaging. Nat. Biotechnol.

[6] Joo C., Balci H., Ishitsuka Y., Buranachai C., Ha T. (2008). Advances in single-molecule fluorescence methods for molecular biology. Annu. Rev. Biochem.

[7] Weiss S. (1999). Fluorescence spectroscopy of single biomolecules. Science.

[8] Weiss S. (2000). Measuring conformational dynamics of biomolecules by single molecule fluorescence spectroscopy. Nat. Struct. Biol.

[9] Roy R., Hohng S., Ha T. (2008). A practical guide to single-molecule FRET. Nat. Methods.

[10] Sorokina M., Koh H.-R., Patel S.S., Ha T. (2009). Fluorescent lifetime trajectories of a single fluorophore reveal reaction intermediates during transcription initiation. J. Am. Chem. Soc.

[11] Muschielok A., Andrecka J., Jawhari A., Brückner F., Cramer P., Michaelis J. (2008). A nano-positioning system for macromolecular structural analysis. Nat. Methods.

[12] Kügel W., Muschielok A., Michaelis J. (2012). Bayesian-inference-based fluorescence correlation spectroscopy and single-molecule burst analysis reveal the influence of dye selection on DNA hairpin dynamics. ChemPhysChem.

[13] Antonik M., Felekyan S., Gaiduk A., Seidel C.A.M. (2006). Separating structural heterogeneities from stochastic variations in fluorescence resonance energy transfer distributions via photon distribution analysis. J. Phys. Chem. B.

[14] Nir E., Michalet X., Hamadani K.M., Laurence T.A., Neuhauser D., Kovchegov Y., Weiss S. (2006). Shot-noise limited single-molecule FRET histograms: Comparison between theory and experiments. J. Phys. Chem. B.

[15] Sisamakis E., Valeri A., Kalinin S., Rothwell P.J., Seidel C.A.M. (2010). Accurate single-molecule FRET studies using multiparameter fluorescence detection. Methods.

[16] Tomov T.E., Tsukanov R., Masoud R., Liber M., Plavner N., Nir E. (2012). Disentangling subpopulations in single-molecule FRET and ALEX experiments with photon distribution analysis. Biophys. J.

[17] Kudryavtsev V., Sikor M., Kalinin S., Mokranjac D., Seidel C.A., Lamb D.C. (2012). Combining MFD and PIE for accurate single-pair Förster resonance energy transfer measurements. ChemPhysChem.

[18] Andrew P., Barnes W.L. (2000). Förster energy transfer in an optical microcavity. Science.

[19] Hopmeier M., Guss W., Deussen M., Göbel E.O., Mahrt R.F. (1999). Enhanced dipole-dipole interaction in a polymer microcavity. Phys. Rev. Lett.

[20] Fujiwara H., Sasaki K., Masuhara H. (2005). Enhancement of Förster energy transfer within a microspherical cavity. ChemPhysChem.

[21] Weyl H. (1919). Ausbreitung elektromagnetischer Wellen über einem ebenen Leiter. Ann. der Physik.

[22] Purcell E.M. (1946). Spontaneous emission probabilities at radio frequencies. Phys. Rev.

[23] Chance R.R., Prock A., Silbey R. (1978). Molecular fluorescence and energy transfer near interfaces. Adv. Chem. Phys.

